# Research Progress on the Treatment of Renal Injury with Esculetin: Multi-Target Pharmacological Mechanism and Clinical Translation Prospect

**DOI:** 10.3390/ijms27125465

**Published:** 2026-06-17

**Authors:** Rujie Zhou, Jianglong Chen, Bin Xia, Meijia Chen, Yu Zhu, Guang Li

**Affiliations:** 1 College of Pharmacy, Heilongjiang University of Chinese Medicine, Harbin 150006, Chinacjl2913183067@163.com (J.C.);; 2 Yunnan Key Laboratory of Southern Medicine Utilization, Yunnan Branch of Institute of Medicinal Plant Development, Chinese Academy of Medical Sciences & Peking Union Medical College, Jinghong 666100, China

**Keywords:** esculetin, kidney injury, pharmacokinetics, toxicity, oxidative stress, inflammatory response, ferroptosis, renal fibrosis

## Abstract

Kidney injury is a major clinical syndrome that can arise from nephrotoxins, ischemia–reperfusion, metabolic disease, infection, and immune dysregulation and can progress from acute kidney injury (AKI) to chronic kidney disease (CKD). Esculetin, a natural 6,7-dihydroxycoumarin derived from Cortex Fraxini, has antioxidant, anti-inflammatory, mitochondrial regulatory, anti-apoptotic, anti-ferroptotic, and anti-fibrotic activities. Preclinical studies report renoprotection in cisplatin-induced AKI, diabetes complicated by ischemia–reperfusion-induced AKI, and adenine-induced chronic renal injury, with changes in Nrf2/HO-1, NF-kappaB/MAPK, PINK1/Parkin-associated mitophagy, endoplasmic reticulum stress, regulated cell death, and fibrotic signaling. However, the evidence is based on a small number of heterogeneous cell and rodent studies, direct molecular targets remain uncertain, and no human studies have validated efficacy, dosing, or safety. Low oral bioavailability, rapid conjugative metabolism, limited long-term toxicology, and the absence of pharmacokinetic–pharmacodynamic relationships are major barriers to translation. This review critically synthesizes the renal evidence for esculetin and identifies the experimental, pharmaceutical, and clinical studies required to determine whether it can progress from a promising multi-target natural product to a renoprotective therapeutic candidate.

## 1. Introduction

Kidney injury is an important pathological basis in the occurrence and progression of renal disease and may manifest as AKI, CKD progression, or AKI-to-CKD transition [1,2,3]. According to Kidney Disease: Improving Global Outcomes (KDIGO), AKI is diagnosed by an increase in serum creatinine of at least 0.3 mg/dL within 48 h, an increase to at least 1.5 times baseline within 7 days, or urine output below 0.5 mL/kg/h for at least 6 h [4]. CKD is defined as abnormalities of kidney structure or function that persist for at least 3 months and have implications for health [5]. CKD affected an estimated 697.5 million people worldwide in 2017, illustrating the scale of chronic renal disease and the importance of preventing progression after acute injury [6]. AKI is commonly induced by ischemia–reperfusion, nephrotoxic drugs, infection, sepsis, contrast exposure, and other insults [1,7,8]. Current clinical management remains dominated by removal of precipitating factors, optimization of hemodynamics, correction of volume and electrolyte disorders, avoidance of nephrotoxins, and renal replacement therapy when necessary [7,9]. However, these strategies are largely supportive, and mechanism-based interventions remain an unmet need [9,10].

AKI is not always completely reversible. When tubular repair is incomplete or inflammation persists, acute injury may progress toward CKD and increase the risk of chronic renal functional decline [11,12]. At the mechanistic level, kidney injury involves tubular epithelial cell damage, oxidative stress, inflammation, mitochondrial dysfunction, apoptosis, ferroptosis, endoplasmic reticulum stress, and renal interstitial fibrosis [13,14]. Nuclear factor erythroid 2-related factor 2 (Nrf2) is a stress-responsive transcription factor that is normally sequestered by Keap1 and targeted for ubiquitin-dependent degradation. Oxidative or electrophilic stress stabilizes Nrf2, promotes its nuclear translocation, and induces antioxidant response element-dependent genes that support redox homeostasis [15,16]. Nrf2-related defense is therefore an important adaptive response in renal oxidative injury, although its biological effect depends on cell type, timing, and injury context [15,16]. Endoplasmic reticulum stress is also increasingly recognized as a hub connecting protein-folding disturbance, mitochondrial dysfunction, programmed cell death, and inflammation during AKI [17,18].

Natural products have attracted increasing attention in kidney injury research because many exert multi-target effects across oxidative stress, inflammation, cell death, and mitochondrial homeostasis [19,20]. Representative compounds such as resveratrol, curcumin, quercetin, and berberine have shown renoprotective activity in experimental AKI models through overlapping redox, inflammatory, mitochondrial, autophagic, or ferroptotic mechanisms [21,22,23,24]. Esculetin, also known as aesculetin, is a natural 6,7-dihydroxycoumarin obtained from Cortex Fraxini, including the bark of Fraxinus rhynchophylla Hance, Fraxinus chinensis Roxb., and related Fraxinus species [25,26]. Compared with these more extensively studied natural products, esculetin is a structurally simple coumarin scaffold with documented renal tissue exposure and a growing set of kidney-specific studies [25,27,28]. However, no head-to-head study demonstrates that esculetin is superior to other nephroprotective natural compounds; its relevance lies in the convergence of a defined chemical scaffold, renal exposure, and reported activity across several complementary renal injury models.

Current experimental evidence suggests that esculetin has renoprotective activity, but the robustness of this evidence is limited. The available kidney-focused literature consists of a small number of cell and rodent studies with substantial differences in species, injury trigger, dose, timing, and outcome measures [29,30,31,32]. The cisplatin mouse model primarily represents acute proximal tubular toxic injury, whereas the adenine model produces crystal-associated tubulointerstitial injury and progressive fibrosis; these models are complementary but are not interchangeable and do not fully reproduce the heterogeneity of human AKI-to-CKD transition [29,30]. The diabetes plus ischemia–reperfusion rat model adds a clinically relevant metabolic comorbidity but may not generalize to non-diabetic ischemic AKI [31,32]. A critical synthesis is therefore needed to distinguish reproducible pharmacodynamic responses from model-specific associations and untested mechanistic hypotheses.

Nevertheless, research on esculetin for kidney injury remains mainly at the cellular and animal experimental stage. Its direct molecular targets, dose–response relationship, pharmacokinetic behavior under kidney dysfunction, long-term safety, and clinical translational value have not yet been fully clarified [25,27,33]. Therefore, this review first outlines the major pathological mechanisms of kidney injury and then summarizes the source, chemical characteristics, pharmacological basis, pharmacokinetics, safety, and renoprotective effects of esculetin. Particular emphasis is placed on its upstream and downstream mechanisms in oxidative stress, inflammatory signaling, mitochondrial quality control, endoplasmic reticulum stress, apoptosis, ferroptosis, and renal fibrosis.

## 2. Major Pathological Mechanisms of Kidney Injury

The occurrence and progression of kidney injury represent an interconnected network rather than a series of independent mechanisms. Oxidative stress and mitochondrial dysfunction amplify inflammatory signaling and regulated cell death, while persistent tubular injury, immune-cell recruitment, and maladaptive repair promote fibroblast activation and extracellular matrix deposition [11,34,35,36,37,38,39]. This interaction is especially important when interpreting esculetin because a change in one downstream biomarker may reflect a secondary consequence of another pathway rather than direct target engagement. Accordingly, the evidence below is evaluated by whether esculetin-associated changes were experimentally measured, causally validated, or inferred from biological plausibility.

### 2.1. Oxidative Stress and Renal Tubular Epithelial Cell Injury

Oxidative stress is an important mechanism in kidney injury and is characterized by increased reactive oxygen species (ROS), reduced antioxidant defense, and lipid peroxidation [13,35]. Renal tubular epithelial cells are metabolically active and are particularly vulnerable to ischemia, hypoxia, nephrotoxins, and high-glucose stimulation [35,36]. Excess ROS can collapse mitochondrial membrane potential, reduce ATP production, enhance lipid peroxidation, and promote DNA damage [13,38]. When oxidative stress persists beyond the acute phase, it can also support fibrotic remodeling. TGF-beta/Smad signaling can induce NOX4-derived ROS in kidney fibroblasts, reinforcing myofibroblast activation and extracellular matrix production, while persistent mitochondrial dysfunction provides an additional source of ROS during maladaptive repair [40,41,42]. In cisplatin-induced AKI, esculetin reduced MDA and 4-HNE and restored GSH [29]. These findings demonstrate an antioxidant phenotype, but they do not establish whether esculetin directly targets NOX4, TGF-beta/Smad signaling, or mitochondrial ROS production.

### 2.2. Inflammatory Responses and Immune-Cell Infiltration

Inflammatory responses occur throughout the onset, progression, and abnormal repair of kidney injury [34,43]. Injured tubular epithelial cells and local immune cells release cytokines and chemokines such as TNF-alpha, IL-1beta, IL-6, MCP-1, CXCL1, and CXCL10, while adhesion molecules including ICAM-1, VCAM-1, and E-selectin facilitate leukocyte recruitment [29,34,44]. Persistent inflammation aggravates tubulointerstitial damage and promotes fibroblast activation and extracellular matrix deposition [11,37]. In cisplatin-induced AKI, esculetin reduced cytokines, chemokines, and adhesion molecule expression [29]. These changes would be expected to limit neutrophil and monocyte recruitment and thereby reduce inflammatory amplification; however, direct quantification of infiltrating immune-cell subsets and their relationship to long-term renal outcomes remains limited.

### 2.3. Mitochondrial Dysfunction and Energy Metabolism Disorder

Mitochondria are the core organelles for energy metabolism in renal tubular epithelial cells, and maintenance of mitochondrial homeostasis is essential for tubular function [36]. During kidney injury, ischemia, hypoxia, toxic drugs, and high-glucose conditions can induce mitochondrial membrane potential loss, excessive ROS generation, and ATP depletion [31,36,37,45,46]. Primary studies have shown that PINK1/Parkin-dependent mitophagy removes damaged mitochondria and helps maintain mitochondrial quality control during AKI [47,48,49]. In a diabetic rat model of ischemia–reperfusion-induced AKI, esculetin increased PINK1, Parkin, and LC3B and decreased p62, together with improved renal injury markers [31]. These are experimentally observed mitophagy-associated changes, but enhanced mitophagic flux and pathway necessity require confirmation using flux inhibitors, genetic perturbation, or direct mitochondrial functional assays. The diabetic comorbidity also limits direct generalization to non-diabetic ischemic AKI.

### 2.4. Apoptosis, Ferroptosis, and Endoplasmic Reticulum Stress

Renal tubular epithelial cell death is a key event in kidney injury, and common regulated forms include apoptosis, necroptosis, and ferroptosis [14,50,51]. Apoptosis is associated with a shift in the Bax/Bcl-2 balance, caspase activation, and mitochondrial pathway signaling [14,50]. Necroptosis is a kinase-regulated lytic process involving RIPK1, RIPK3, and MLKL, whereas ferroptosis is an iron-dependent form of cell death driven by phospholipid peroxidation and is mechanistically distinct from necroptosis [51,52,53]. Ferroptosis is governed not only by the SLC7A11/GSH/GPX4 defense axis but also by ACSL4-dependent enrichment of polyunsaturated phospholipids, iron uptake and storage, and GPX4-parallel defense systems such as FSP1 [51,52,54,55,56,57,58]. In cisplatin-induced AKI, esculetin reduced ACSL4 and TFR1, preserved GPX4 and SLC7A11, and decreased lipid peroxidation-related injury [29]. This supports an anti-ferroptotic association, but definitive validation should include ferroptosis-specific rescue, renal iron quantification, oxidized phospholipid lipidomics, and assessment of FSP1-related defense.

### 2.5. Renal Fibrosis and AKI-to-CKD Transition

Renal fibrosis is a common pathological endpoint of CKD progression and an important manifestation of maladaptive repair after AKI [11,37,59,60,61]. Persistent tubular injury, inflammation, hypoxia, oxidative stress, and cell-cycle arrest can activate resident fibroblasts and pericyte-derived stromal cells and promote myofibroblast accumulation [62,63]. Tubular epithelial cells may undergo partial epithelial–mesenchymal transition-related changes and secrete profibrotic signals without necessarily becoming the dominant myofibroblast source [64]. Metabolic reprogramming in tubular, immune, and stromal cells can further sustain chronic inflammation and extracellular matrix deposition [65,66]. In adenine-induced renal injury, esculetin reduced inflammation, oxidative stress, tubular dilation, and collagen deposition and inhibited the EGFR/SRC/PI3K/AKT/NF-kappaB axis [30]. These findings establish an anti-fibrotic phenotype in a crystal-associated chronic tubulointerstitial injury model, but direct effects on resident fibroblasts, pericytes, TGF-beta/Smad signaling, and fibrotic metabolic reprogramming remain untested.

## 3. Source, Chemical Characteristics, and Pharmacological Basis of Esculetin

**Figure 1:**
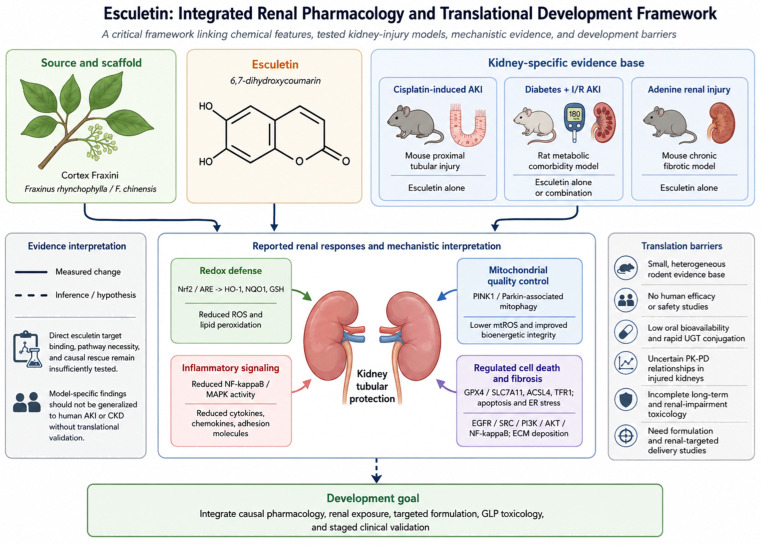
Integrated renal pharmacology and translational development framework for esculetin. The 6,7-dihydroxycoumarin scaffold is linked to the kidney injury models in which esculetin has been evaluated and to reported changes in Nrf2-related redox defense, NF-kappaB/MAPK signaling, PINK1/Parkin-associated mitophagy, regulated cell death, and fibrotic signaling. Solid arrows indicate experimentally measured changes, whereas dashed arrows indicate mechanistic inference or future validation needs. Major translation barriers are also shown.

Esculetin is a natural coumarin-derived bioactive component from Cortex Fraxini and related medicinal plants [25,26]. For kidney injury research, its most relevant features are the 6,7-dihydroxycoumarin scaffold, documented renal tissue exposure, and reported pharmacodynamic effects on tubular injury, oxidative stress, inflammation, mitochondrial quality control, regulated cell death, and fibrosis [27,28]. The broad non-renal pharmacology of esculetin provides mechanistic plausibility but should not be treated as evidence of renal efficacy. A kidney-focused evaluation must instead consider renal cell types, disease context, exposure, and the strength of causal pathway validation (Figure 1).

### 3.1. Source and Chemical Characteristics of Esculetin

Esculetin, also known as 6,7-dihydroxycoumarin, is a natural dihydroxycoumarin compound [25]. It is mainly derived from Cortex Fraxini, the dried bark of Fraxinus rhynchophylla Hance, Fraxinus chinensis Roxb., and related Fraxinus species of the Oleaceae family [25,67]. Cortex Fraxini contains several coumarins, among which esculin and esculetin are representative constituents [67]. Esculin is the glycoside form of esculetin, whereas esculetin is the aglycone, and pharmacokinetic studies show that esculin can be metabolized to esculetin in vivo [68]. This relationship is relevant to renal pharmacology because exposure may arise from administered esculetin, metabolic conversion of esculin, or both.

Chemically, esculetin is a coumarin nucleus bearing two hydroxyl groups. Its chemical name is 6,7-dihydroxycoumarin, its molecular formula is C_9_H_6_O_4_, and its relative molecular mass is approximately 178.14. The phenolic hydroxyl groups at positions 6 and 7 in the esculetin structure are closely related to its free radical–scavenging activity and redox-modulating effects [67,69]. Compared with esculin, which contains a glycosyl moiety, esculetin exists as an aglycone and may therefore differ in lipophilicity, absorption and transport, metabolic patterns, and bioavailability [68]. Because esculetin has a clear structure, a relatively well-defined source, and a solid pharmacological research foundation, it has become an important subject in studies of active constituents of Cortex Fraxini and in the development of natural coumarin compounds [68].

### 3.2. Major Pharmacological Effects of Esculetin

The two adjacent phenolic hydroxyl groups at positions 6 and 7 are central to the chemical and pharmacological behavior of esculetin. They support radical scavenging and redox modulation but also create sites for rapid phase II conjugation, which may limit systemic and renal exposure [25,67,70]. In kidney-focused studies, the most consistently measured pharmacodynamic responses include reductions in SCr, BUN, tubular injury markers, cytokines, lipid peroxidation, and collagen deposition, together with changes in mitophagy- and cell-death-related proteins [29,30,31]. However, direct binding targets in renal cells have not been established, and the relationship between the chemical scaffold, target engagement, and nephroprotection remains largely inferential.

The renal evidence for esculetin is strongest in tubular-dominant injury models. In cisplatin-induced AKI, esculetin changed tubular injury markers, inflammatory mediators, oxidative stress indices, endoplasmic reticulum stress proteins, and apoptosis- and ferroptosis-associated proteins [29]. In diabetes complicated by ischemia–reperfusion injury, esculetin changed renal function and PINK1/Parkin-associated mitophagy readouts [31]. In adenine-induced renal injury, it reduced fibrotic remodeling and phosphorylation within the EGFR/SRC/PI3K/AKT/NF-kappaB axis [30]. By contrast, pharmacodynamic effects in podocytes, mesangial cells, glomerular endothelial cells, and renal interstitial fibroblasts remain insufficiently characterized.

Broad activities reported in non-renal systems, including antidiabetic, antitumor, antibacterial, and immunomodulatory effects, may be relevant to kidney disease but cannot substitute for direct renal evidence [25,67,69]. Current kidney studies do not include head-to-head comparisons with other natural compounds, systematic structure-activity analyses, or disease-specific therapeutic windows. Future work should determine whether modification of the 6,7-dihydroxycoumarin scaffold can preserve renal pharmacology while improving metabolic stability and whether esculetin has cell-type-specific effects within the injured kidney.

## 4. Pharmacokinetic Studies of Esculetin

Pharmacokinetic research clarifies drug absorption, distribution, metabolism, and excretion in vivo and is an important basis for evaluating the druggability and clinical translational value of esculetin [25,27]. Although pharmacokinetic studies on esculetin remain limited, available investigations have examined plasma concentration, oral bioavailability, tissue distribution, biliary excretion, and metabolic transformation [27,70]. Current evidence shows that esculetin can enter the circulation after oral administration and can be detected in liver and kidney tissues [28]. However, its oral bioavailability is relatively low, suggesting that absorption, metabolism, and formulation-related factors may limit its in vivo pharmacological effects [27,71].

### 4.1. Absorption and Oral Bioavailability

As a natural small-molecule coumarin compound, the absorption of esculetin after oral administration directly affects systemic exposure and renal pharmacological efficacy [25,27]. Rat pharmacokinetic studies report mean oral bioavailability of approximately 15.6–20.3%, indicating measurable absorption but limited overall exposure [72]. Low oral bioavailability probably reflects a combination of limited solubility or intestinal permeability and presystemic metabolism. The phenolic hydroxyl groups of esculetin favor phase II conjugation, and UGT1A6 and UGT1A9 catalyze 7-O-glucuronidation in human liver microsomes [70]. However, the contributions of intestinal UGTs, enterocyte metabolism, transporter-mediated efflux, and first-pass hepatic extraction have not been quantitatively separated. Future studies should combine permeability assays, intestinal perfusion, portal and systemic sampling, and metabolite-resolved pharmacokinetics to identify the dominant exposure-limiting step under healthy and kidney-injury conditions.

### 4.2. In Vivo Distribution Characteristics

The tissue distribution of esculetin determines whether it can reach target organs such as the kidney and exert protective effects [28]. Kim et al. [28] established an HPLC-UV method to detect esculetin in rat plasma and tissues and evaluated changes in plasma concentration and tissue distribution after oral administration. The study showed detectable plasma exposure after oral administration, with a peak plasma concentration of approximately 173.3 ng/mL and an elimination half-life of approximately 45 min, indicating rapid absorption but a relatively short residence time in vivo [28]. In terms of tissue distribution, esculetin was detectable in the liver and kidney after oral administration and remained detectable in both tissues at 180 min, suggesting that the liver and kidney may be important exposure tissues [28]. This finding is meaningful for kidney injury research because the ability of esculetin to reach renal tissue is an important pharmacokinetic basis for its renoprotective effects [28]. However, detailed studies on the distribution of esculetin in the renal cortex, renal medulla, renal tubular epithelial cells, or renal interstitial cells remain scarce, and current evidence is insufficient to fully explain exposure differences among renal regions and cell types [28]. Therefore, future studies may combine techniques such as tissue mass spectrometry imaging, microdialysis, or targeted quantitative analysis to further define the spatiotemporal distribution of esculetin in renal tissue [28,73].

### 4.3. Metabolism and Excretion

After entering the body, esculetin undergoes multiple metabolic transformations, with phase II conjugation recognized as a major pathway [25,70]. Zhu et al. showed that esculetin forms a 7-O-glucuronide in human liver microsomes and identified UGT1A6 and UGT1A9 as major responsible isoforms [70]. Recent metabolomic studies in rats also identified hydroxylation, hydrogenation, dehydroxylation, glucuronidation, sulfation, and methylation products [74]. These findings support extensive hepatic conjugation, but intestinal metabolism and the pharmacological activity of circulating or renal metabolites remain poorly defined. Because kidney injury can alter transporter function, protein binding, metabolism, and excretion, studies should determine whether esculetin or its conjugates accumulate and whether metabolites contribute to efficacy or toxicity.

With respect to excretion, biliary excretion may be an important route of esculetin disposition in vivo [73]. Tsai et al. [73] used simultaneous blood and biliary microdialysis in rats and found that bile concentrations were higher than blood concentrations, suggesting marked biliary transport and excretion. Differences in the distribution and elimination half-lives of esculetin between blood and bile indicate that its in vivo clearance may be influenced by both circulatory transport and hepatobiliary excretion [74]. For kidney injury research, the relative contribution of hepatobiliary and renal excretion should be clarified because renal dysfunction may alter exposure and safety [70,73]. At present, studies on urinary excretion, fecal excretion, and excretion kinetics under kidney injury conditions remain insufficient.

### 4.4. Pharmacokinetic Limitations and Formulation Improvement

Although esculetin has multiple pharmacological activities, including antioxidant, anti-inflammatory, and anti-apoptotic effects, its relatively low oral bioavailability, rapid in vivo clearance, and marked metabolic transformation may limit stable pharmacological efficacy. Existing studies suggest that the mean oral bioavailability of esculetin is approximately 19%, and this low bioavailability is closely associated with insufficient absorption and extensive glucuronidation metabolism. Therefore, improving solubility, absorption efficiency, systemic exposure, and target-tissue delivery is an important issue for its further development.

Formulation strategies have begun to address the exposure limitations of esculetin [71]. Esculetin-loaded mixed micelles increased oral bioavailability by approximately 3.06-fold and prolonged half-life by approximately 1.45-fold compared with free esculetin [71]. Beyond mixed micelles, lipid nanoparticles, polymeric nanoparticles, prodrugs, and inflammation-responsive carriers could improve solubility, protect esculetin from intestinal or hepatic metabolism, and alter tissue distribution. Renotropic delivery systems may also exploit particle size and charge, proximal tubular uptake pathways, or injury-associated targets such as KIM-1 to enrich drug exposure in damaged tubules. These concepts remain untested for esculetin in kidney injury. Future studies should compare kidney-to-plasma exposure, target-cell uptake, metabolite profiles, efficacy, and chronic safety rather than assuming that higher systemic exposure will necessarily improve renal benefit.

## 5. Toxicity and Safety Studies of Esculetin

As a natural coumarin compound, esculetin has shown multiple pharmacological activities, including antioxidant, anti-inflammatory, and tissue-protective effects. Nevertheless, further development and application must be based on systematic toxicity and safety evaluation [25,75]. Available data indicate that the acute toxicity of esculetin is relatively low, but information on long-term toxicity, genotoxicity, reproductive toxicity, safety under kidney injury conditions, and safety in clinical populations remains insufficient [33]. Because patients with kidney injury often have reduced renal function, weakened metabolic and excretory capacity, and concomitant use of multiple drugs, it is important to evaluate the safety window of esculetin in the context of kidney injury [27,33,70].

### 5.1. Acute Toxicity Studies

Acute toxicity studies are the first step in evaluating the basic safety of esculetin and provide a basis for subsequent dose design and determination of the safety range [33]. Assessment data from the European Medicines Agency indicate that esculetin derived from horse chestnut bark has an intraperitoneal LD50 of 1450 mg/kg in mice and an oral LD50 greater than 2000 mg/kg, suggesting relatively low acute toxicity. The same report also states that the intraperitoneal LD50 of esculin is 1900 mg/kg, indicating that esculetin and related coumarin components generally have favorable safety at the acute toxicity level. However, existing acute toxicity data mainly originate from early animal studies, and differences in source, purity, route of administration, and experimental conditions may affect the comparability of toxicity results [33]. Therefore, future studies should follow modern toxicological evaluation standards and systematically observe mortality, behavioral changes, body-weight variation, hematological and biochemical indices, and pathological changes in major organs after a single dose of esculetin [33].

### 5.2. Subchronic Toxicity and Long-Term Safety

Kidney injury, especially chronic kidney injury and renal fibrosis, often requires long-term intervention; therefore, evaluation of subchronic toxicity and long-term safety is important [11,33]. Publicly available data show that repeated-dose toxicity information for esculetin remains scarce, and the European Medicines Agency assessment report also notes a lack of repeated-dose toxicity studies [33]. This limitation restricts evaluation of the safe range for long-term esculetin administration, accumulation risk, and potential target-organ toxicity. In kidney injury studies, long-term administration experiments should focus on serum creatinine, blood urea nitrogen, urinary protein, hepatic and renal function, complete blood counts, and pathological changes in major organs [7,33]. Chronic decline in renal function may also alter the in vivo exposure of esculetin and its metabolites [27,70].

### 5.3. Cytotoxicity Studies

Cytotoxicity studies help clarify the safe concentration range of esculetin in different cellular models and provide a basis for dose selection in in vitro kidney injury experiments [25,75]. Existing studies have shown that esculetin can exert anti-inflammatory, antioxidant, and cytoprotective effects within certain concentration ranges, but at higher concentrations or in specific cell types it may inhibit proliferation or induce cell death [67,75]. In tumor-cell studies, esculetin induces apoptosis through MAPK- and caspase-related pathways, suggesting that its cellular effects are dose-dependent and cell type-specific [75]. Therefore, in kidney injury-related studies, the safe concentration ranges of esculetin should be evaluated separately in renal tubular epithelial cells, podocytes, mesangial cells, endothelial cells, and renal interstitial fibroblasts [13,75]. Under high glucose, hypoxia/reoxygenation, cisplatin, or inflammatory stimulation, the margin between cytoprotective and potentially cytotoxic concentrations requires further clarification [29,31].

### 5.4. Safety Issues in the Context of Kidney Injury

The kidney is not only an important target organ in which esculetin may exert protective effects, but also a key organ for drug excretion and toxicity assessment [27,28]. Existing tissue distribution studies show that esculetin can be detected in renal tissue after oral administration, indicating a pharmacokinetic basis for reaching the kidney [28]. However, reduced renal function may alter drug clearance and metabolite excretion, thereby affecting systemic exposure and safety [70,73]. During AKI, CKD, and AKI-to-CKD transition, systemic inflammation, oxidative stress, and tubular transport function may also change [11,43]. Therefore, future studies should conduct pharmacodynamic, pharmacokinetic, and toxicological evaluations simultaneously in kidney injury models [27,33].

### 5.5. Limitations of Current Toxicity Studies

Overall, esculetin currently has some acute toxicity data, but systematic toxicity studies remain clearly insufficient. Existing evidence does not define a no-observed-adverse-effect level, an exposure-based safety margin, or the consequences of impaired renal function. Translational development will require a staged preclinical safety package aligned with small-molecule regulatory expectations, including good laboratory practice repeated-dose toxicity with toxicokinetics and histopathology, safety pharmacology, genotoxicity, reproductive and developmental toxicity when appropriate, impurity and formulation characterization, and evaluation of drug–drug interactions. Because one proposed application is prevention of cisplatin nephrotoxicity, studies must also confirm that esculetin does not reduce anticancer efficacy or alter cisplatin exposure. These requirements should be completed before first-in-human evaluation.

## 6. Protective Effects of Esculetin Against Different Types of Kidney Injury

Experimental studies have evaluated esculetin in several representative kidney injury models, including cisplatin-induced AKI, diabetes complicated by ischemia–reperfusion injury, and adenine-induced chronic renal injury or fibrotic progression [29,30,31,32]. Table 1 summarizes the experimental model, dose, administration route, evaluated biomarkers, reported mechanisms, principal findings, and major limitations. Because the studies differ substantially in design and disease context, their findings should be compared as complementary model-specific evidence rather than pooled as proof of a single universal renoprotective mechanism.

### 6.1. Esculetin and Drug-Induced Kidney Injury

Drug-induced kidney injury is an important type of AKI, with causes including cisplatin, aminoglycosides, non-steroidal anti-inflammatory drugs, and contrast agents [76]. Cisplatin nephrotoxicity is particularly relevant because cisplatin is transported into proximal tubular cells in part by organic cation transporter 2 (OCT2), accumulates in the kidney, and triggers DNA damage responses, p53-associated apoptosis, mitochondrial stress, ROS generation, inflammation, endoplasmic reticulum stress, and ferroptosis [77,78,79]. Kim et al. administered esculetin at 40 mg/kg/day by oral gavage for 4 days before cisplatin challenge in male C57BL/6 mice [29]. Esculetin reduced SCr, BUN, histological injury, NGAL, KIM-1, cytokines, chemokines, adhesion molecules, MDA, 4-HNE, UPR markers, p53, cleaved caspase-3, ACSL4, and TFR1 and preserved GSH, GPX4, and SLC7A11 [29]. Reductions in chemokines and adhesion molecules are consistent with less leukocyte recruitment and inflammatory amplification. The study provides broad phenotypic evidence, but it used one preventive dose, did not test tumor-bearing animals, did not measure OCT2-dependent platinum accumulation, and did not use pathway-specific rescue or long-term fibrosis endpoints.

### 6.2. Esculetin and Diabetes-Related Kidney Injury

Diabetes-related kidney injury is associated with chronic hyperglycemia, glucolipid metabolic disturbance, inflammation, oxidative stress, and reduced mitochondrial reserve [13,31]. In diabetic rats subjected to bilateral renal ischemia–reperfusion injury, oral esculetin pretreatment reduced plasma creatinine, BUN, KIM-1, and oxidative injury and changed PINK1, Parkin, LC3B, and p62 expression [31]. A later combination study reported additional benefit when esculetin was administered with phloretin [32]. These findings support investigation of esculetin in metabolically vulnerable kidneys, but the combination study cannot isolate the contribution of esculetin, and neither model establishes efficacy in chronic diabetic nephropathy or in non-diabetic AKI.

### 6.3. Esculetin and Ischemia/Reperfusion-Induced Kidney Injury

Ischemia–reperfusion-induced kidney injury commonly occurs in shock, kidney transplantation, major surgery, and diabetes complicated by AKI [1,35]. Ischemia causes ATP depletion and tubular energetic failure, whereas reperfusion produces ROS bursts, inflammatory signaling, and mitochondrial damage [35,36]. PINK1/Parkin-dependent mitophagy has a protective role in experimental ischemic AKI by removing damaged mitochondria [47,48,49]. Esculetin-associated improvement has so far been demonstrated primarily in diabetic ischemia–reperfusion models [31,32]. This is clinically relevant because diabetes increases AKI susceptibility, but it also limits generalization. Standalone non-diabetic ischemia–reperfusion studies, delayed-treatment designs, and transplant-relevant models are needed before concluding that esculetin is broadly effective against ischemic AKI.

### 6.4. Esculetin and Chronic Kidney Injury/Renal Fibrosis

Progression of chronic kidney injury is closely related to persistent inflammation, oxidative stress, tubulointerstitial injury, fibroblast activation, and extracellular matrix deposition [11,37,61]. Resident fibroblasts and pericyte-derived stromal cells are major contributors to myofibroblast accumulation, while partial epithelial–mesenchymal transition-related changes, cell-cycle arrest, and metabolic reprogramming in tubular and immune cells can sustain profibrotic signaling [62,63,64,66]. In an adenine-induced model, esculetin reduced SCr, BUN, tubular dilation, inflammatory infiltration, and collagen deposition and inhibited phosphorylation of EGFR, SRC, PI3K, AKT, and p65 [30]. These findings demonstrate an anti-fibrotic phenotype, but the adenine model is a crystal-associated chronic tubulointerstitial injury model rather than a complete representation of clinical AKI-to-CKD transition. Direct effects on resident fibroblasts, pericytes, epithelial plasticity, TGF-beta/Smad signaling, and fibrotic metabolic reprogramming remain to be tested.

## 7. Mechanisms of Esculetin in the Prevention and Treatment of Kidney Injury

The effects of esculetin in kidney injury are best understood as a multi-level protective network rather than as a single-target action [25,29,30]. To avoid conflating biomarker association with target validation, the mechanisms in this section are classified as experimentally demonstrated responses, mechanistic inferences, or future hypotheses. Demonstrated responses include esculetin-associated changes in renal function, tubular injury, oxidative stress, cytokines, mitophagy-related proteins, cell-death-related proteins, and fibrotic signaling in specific models [29,30,31]. Mechanistic inferences include the interpretation that these changes reflect causal activation or inhibition of Nrf2/HO-1, NF-kappaB/MAPK, PINK1/Parkin, ferroptosis, or fibrotic pathways. Direct esculetin targets, pathway necessity, and primary versus secondary effects remain future hypotheses that require binding studies, inhibitor rescue, gene perturbation, and multi-omics validation.

### 7.1. Regulation of Oxidative Stress and the Nrf2/HO-1 Antioxidant Pathway

**Figure 2:**
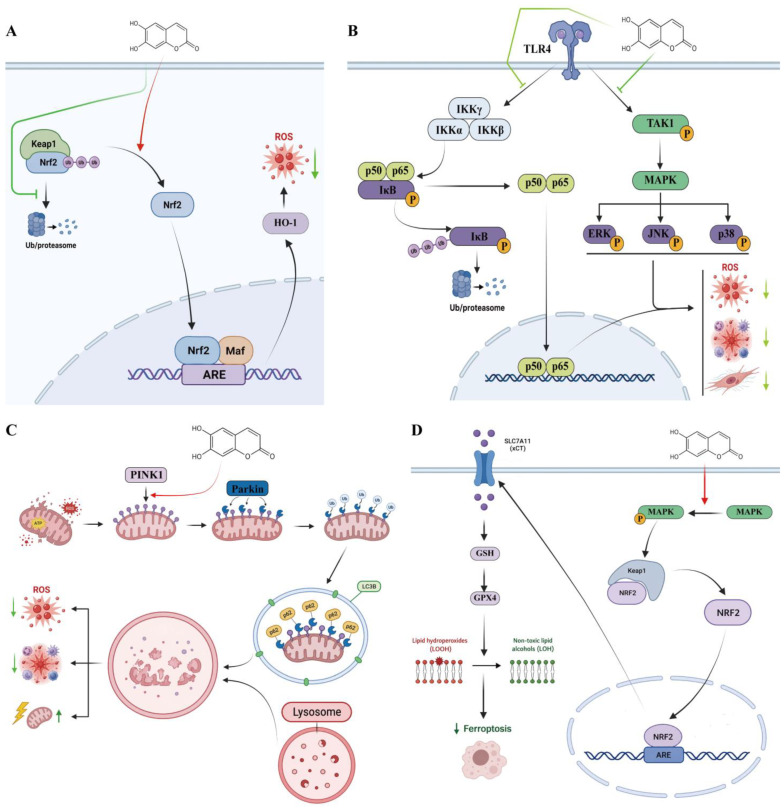
Schematic representation of Esculetin-mediated regulation of multiple kidney injury pathways. (**A**). Esculetin inhibits Keap1-mediated ubiquitination and degradation of Nrf2, promoting its nuclear translocation and heterodimerization with Maf to bind antioxidant response elements (ARE), thereby inducing HO-1 expression, reducing intracellular reactive oxygen species (ROS) levels, and alleviating oxidative stress. (**B**). Esculetin suppresses TLR4-mediated inflammatory signaling by blocking IKK complex activation, preventing IκB phosphorylation and ubiquitination, thereby inhibiting NF-κB p50/p65 nuclear translocation and reducing the expression of pro-inflammatory cytokines (TNF-α, IL-1β, IL-6, MCP-1, etc.). Concurrently, Esculetin inhibits TAK1/MAPK pathway phosphorylation (ERK, JNK, p38), attenuating ROS production and inflammatory cell infiltration. (**C**). Esculetin activates PINK1/Parkin-mediated mitophagy, promoting ubiquitination of damaged mitochondria and LC3B-dependent autophagosome formation. p62 is degraded during this process, facilitating the clearance of damaged mitochondria, reducing mitochondrial ROS generation, maintaining ATP levels, and protecting renal tubular epithelial cell survival. (**D**). Esculetin activates the AMPK/Nrf2/SLC7A11-GSH-GPX4 axis, enhancing cystine uptake and glutathione (GSH) synthesis, maintaining GPX4 activity to convert lipid hydroperoxides into non-toxic lipid alcohols (LOH), thereby suppressing ferroptosis and lipid peroxidation-induced cellular injury. Legend: Red arrows indicate activation signals, and green arrows indicate inhibitory signals.

Oxidative stress is a core upstream event in kidney injury and is characterized by excessive ROS generation, depletion of antioxidant defenses, and lipid peroxidation [13,35]. The Keap1/Nrf2/ARE pathway is a major endogenous antioxidant system: Keap1 promotes Nrf2 degradation under basal conditions, whereas oxidative or electrophilic stress stabilizes Nrf2 and induces genes involved in GSH synthesis, detoxification, and redox control [80]. Esculetin restored GSH and reduced MDA and 4-HNE in cisplatin-induced AKI, demonstrating reinforcement of the renal antioxidant phenotype [29]. However, Nrf2 activation should not be assumed to be uniformly beneficial. Transient, cell-appropriate activation can be protective, whereas prolonged or non-physiological activation may alter metabolic and stress-response programs; the outcome depends on injury phase, cell type, and dose [15,16]. Nrf2 also shows reciprocal redox-sensitive crosstalk with NF-kappaB signaling. Whether Nrf2 is required for esculetin-mediated renal protection has not yet been tested using Nrf2 loss-of-function or pathway-rescue approaches (Figure 2A).

### 7.2. Inhibition of Inflammatory Responses and the NF-κB/MAPK Signaling Pathway

Inflammatory signaling is another major upstream amplifier of kidney injury. Damaged tubular epithelial cells release DAMPs, chemokines, and inflammatory cytokines, which activate innate immune pathways and recruit neutrophils, monocytes, and macrophages [34,44]. At the intracellular level, ROS and receptor-mediated signals such as TLR4 activation can stimulate IKK, promote I-kappaBalpha phosphorylation and degradation, and allow NF-kappaB p65 nuclear translocation, thereby increasing transcription of TNF-alpha, IL-1beta, IL-6, MCP-1, ICAM-1, VCAM-1, COX-2, and iNOS [29,34]. The MAPK family, including ERK, JNK, and p38, also participates in inflammatory amplification, stress responses, apoptosis, and fibrotic remodeling [29,76]. In sepsis-associated and contrast-induced AKI, these inflammatory pathways interact with mitochondrial dysfunction and oxidative stress, further illustrating their broad relevance across different injury triggers (Figure 2B) [81,82]. Esculetin has been shown to reduce renal cytokine production and suppress ERK1/2 and p38 phosphorylation in cisplatin-induced AKI [29]. In adenine-induced renal injury, esculetin also inhibited the EGFR/SRC/PI3K/AKT/NF-kappaB axis, suggesting that its anti-inflammatory effect may extend from acute injury control to chronic fibrosis prevention [30].

### 7.3. Improvement of Mitochondrial Dysfunction and PINK1/Parkin-Mediated Mitophagy

Mitochondria are central organelles for ATP production in renal tubular epithelial cells, and mitochondrial dysfunction is both an upstream driver and a downstream consequence of kidney injury [36,38]. PINK1/Parkin-dependent mitophagy is a major quality-control mechanism in which damaged mitochondria accumulate PINK1, recruit Parkin, undergo ubiquitination, and are delivered to autophagic degradation [48,49]. In diabetic ischemia–reperfusion-induced AKI, esculetin increased PINK1, Parkin, and LC3B and reduced p62 together with improved renal injury markers [31]. These are demonstrated mitophagy-associated changes. Enhanced mitophagic flux, preservation of tubular bioenergetics, and pathway necessity remain mechanistic inferences because flux-blocking, genetic, and direct ATP or mitochondrial respiration studies were not reported (Figure 2C).

### 7.4. Inhibition of Apoptosis and Protection of Renal Tubular Epithelial Integrity

Apoptosis is a major downstream form of tubular epithelial cell loss during AKI. Upstream stimuli such as cisplatin, hypoxia, ROS overload, inflammatory cytokines, and endoplasmic reticulum stress can shift the Bax/Bcl-2 balance toward mitochondrial apoptosis [29,50]. Bax activation increases mitochondrial outer membrane permeability, induces cytochrome c release, activates caspase-9, and subsequently triggers caspase-3-mediated execution of apoptosis [50]. Excessive apoptosis reduces the number of functional tubular epithelial cells, disrupts tubular architecture, and contributes to renal dysfunction [14,50]. Esculetin has been reported to reduce tubular cell death in cisplatin-induced AKI and to regulate apoptosis-related proteins, including Bax, Bcl-2, and cleaved caspase-3 [29]. Mechanistically, the anti-apoptotic effect of esculetin is likely connected to its upstream inhibition of oxidative stress, mitochondrial dysfunction, inflammatory signaling, and CHOP-associated endoplasmic reticulum stress [17,29].

### 7.5. Inhibition of Ferroptosis and Lipid Peroxidation Injury

Ferroptosis is an iron-dependent form of regulated cell death driven by uncontrolled phospholipid peroxidation [51,52]. Its regulation involves iron handling, ACSL4-dependent phospholipid composition, the SLC7A11/GSH/GPX4 axis, and GPX4-parallel defenses such as FSP1 (Figure 2D) [51,52,54,55,56,57,58]. In cisplatin-induced AKI, esculetin reduced ACSL4 and TFR1, preserved GPX4 and SLC7A11, and decreased lipid peroxidation-related injury [29]. These findings support suppression of a ferroptosis-associated phenotype. Definitive demonstration requires ferroptosis-specific rescue, assessment of renal iron pools and FSP1, and lipidomic identification of oxidized phospholipids. The distinction is important because apoptosis, necroptosis, and ferroptosis can coexist in injured tubules and cannot be assigned solely from a small set of protein markers.

### 7.6. Attenuation of Endoplasmic Reticulum Stress and the Unfolded Protein Response

Endoplasmic reticulum stress is activated when unfolded or misfolded proteins accumulate in tubular epithelial cells under toxic, ischemic, oxidative, or metabolic stress [17]. The unfolded protein response initially attempts to restore protein homeostasis through PERK/eIF2alpha/ATF4, IRE1alpha/XBP1, and ATF6 signaling [83]. However, persistent activation converts this adaptive response into a pro-injury program, especially through CHOP induction, calcium imbalance, ROS generation, and crosstalk with mitochondrial apoptosis [18,83]. Recent reviews indicate that ER stress can interact with autophagy, apoptosis, pyroptosis, and ferroptosis during AKI, suggesting that it acts as a central stress-integration hub rather than an isolated pathway [18,83]. Natural products derived from traditional Chinese medicine have also been reviewed as potential ER stress modulators in kidney diseases, supporting the plausibility of targeting this pathway pharmacologically [84]. In cisplatin-induced AKI, esculetin decreased unfolded protein response-related markers and attenuated CHOP-associated endoplasmic reticulum stress [29]. Therefore, ER stress attenuation may serve as an intermediate mechanism through which esculetin reduces mitochondrial damage, apoptosis, and tubular dysfunction [85].

### 7.7. Inhibition of Renal Fibrosis and AKI-to-CKD Transition

Renal fibrosis is a major downstream consequence of maladaptive repair after AKI [86]. Persistent tubular injury, inflammatory-cell infiltration, oxidative stress, hypoxia, cell-cycle arrest, fibroblast activation, and metabolic reprogramming can all contribute to myofibroblast accumulation and extracellular matrix deposition [87,88]. In adenine-induced renal injury, esculetin reduced SCr, BUN, tubular dilation, collagen deposition, and phosphorylation of EGFR, SRC, PI3K, AKT, and p65 [30]. This establishes a model-specific anti-fibrotic phenotype. The interpretation that esculetin directly regulates fibroblasts, pericytes, TGF-beta/Smad signaling, or metabolic remodeling remains an inference that should be tested in cell-type-resolved and pathway-intervention studies.

### 7.8. Multi-Pathway Synergistic Regulation and the Potential Mechanistic Network

Overall, the current renal evidence supports a coordinated pharmacodynamic response rather than a validated single molecular target. Experimentally measured effects include reduced oxidative injury and inflammation, altered mitophagy-associated proteins, suppression of apoptosis- and ferroptosis-associated markers, and reduced fibrotic remodeling in specific rodent models [29,30,31]. Biologically plausible inferences connect these responses to Keap1/Nrf2/ARE, NF-kappaB/MAPK, PINK1/Parkin, PERK/eIF2alpha/ATF4/CHOP, SLC7A11/GSH/GPX4, and EGFR/SRC/PI3K/AKT/NF-kappaB signaling. Future hypotheses include direct esculetin target binding, causal pathway hierarchy, cell-type-specific actions, and interactions among redox signaling, regulated cell death, and fibrosis. These questions should be addressed using inhibitor rescue, gene knockdown or knockout, pathway overactivation, target-engagement assays, and multi-omics validation.

## 8. Limitations of Current Research on Esculetin for the Prevention and Treatment of Kidney Injury

Although esculetin has shown potential in the prevention and treatment of kidney injury, the current evidence remains insufficient to support direct clinical application. Most studies are still based on animal models or cellular experiments, and high-quality clinical evidence is lacking. In addition, differences in dosage, administration timing, model type, and outcome indicators reduce comparability across studies. More recent AKI research highlights the need to integrate mechanistic studies with biomarkers, omics, imaging, and translational validation rather than relying only on conventional renal function indicators.

### 8.1. Kidney Injury Models Remain Insufficiently Systematic

Existing studies on the renoprotective effects of esculetin mainly focus on cisplatin-induced AKI, diabetes complicated by ischemia–reperfusion injury, and adenine-induced renal injury. These models cover drug-induced injury, ischemia–reperfusion injury, and chronic fibrotic progression, but they cannot fully represent the diverse etiologies of clinical kidney injury. For example, the effects of esculetin remain insufficiently investigated in contrast-induced nephropathy, sepsis-associated AKI, immune-mediated glomerulonephritis, and hypertensive kidney injury. Future studies should establish broader and more clinically relevant models to determine the applicable scope of esculetin.

### 8.2. Insufficient Integration of Pharmacodynamics and Pharmacokinetics

Most current studies focus on pharmacological efficacy and molecular mechanisms, whereas the relationship among plasma exposure, renal tissue distribution, metabolites, and therapeutic effects remains underexplored. Esculetin has relatively low oral bioavailability and extensive metabolic transformation, which may affect the stability of its in vivo efficacy. Although esculetin can be detected in renal tissue after oral administration, its effective concentration, retention time, and cellular localization in injured kidney tissue remain unclear. Future pharmacokinetic–pharmacodynamic studies should clarify the dose–exposure–effect relationship of esculetin in different kidney injury states.

### 8.3. Insufficient Safety Research

Some acute toxicity data are available for esculetin, but evaluations of long-term toxicity and special toxicity remain incomplete. For kidney injury prevention and treatment, the safety of long-term administration and use under reduced renal function is a critical issue. At present, systematic toxicological evaluations after long-term administration of esculetin in chronic kidney injury models are lacking, as are safety studies of esculetin in combination with commonly used renoprotective drugs or anticancer agents. Therefore, future studies should systematically investigate acute toxicity, subchronic toxicity, genotoxicity, reproductive toxicity, and toxicity under kidney injury conditions.

### 8.4. Insufficient Evidence for Clinical Translation

At present, evidence supporting esculetin for kidney injury is mainly derived from animal and cellular experiments, and clinical evidence remains lacking. Its effective dose, administration route, therapeutic window, and population-level safety have not yet been established. In addition, low oral bioavailability and rapid metabolism indicate that formulation optimization or structural modification may be required for translational development. Recent non-clinical studies targeting AKI-to-CKD transition suggest that therapeutic development should evaluate not only early tubular injury but also long-term fibrosis, renal function recovery, and chronic transition endpoints. Therefore, advancement toward clinical research should be based on integrated pharmacodynamic, pharmacokinetic, toxicological, and formulation studies.

## 9. Perspectives

Future research on esculetin should first validate efficacy across clinically relevant kidney injury models using standardized doses, therapeutic rather than exclusively preventive timing, both sexes, and long-term functional and fibrotic outcomes. Human kidney organoids and proximal tubule-on-a-chip systems can complement animal studies by testing tubular toxicity, transport, metabolism, and cell-specific responses in human-derived platforms. Single-cell RNA sequencing and spatial transcriptomics can then identify which renal cell populations respond to esculetin, distinguish primary target cells from secondary responders, and map interactions among injured tubules, immune cells, fibroblasts, and pericytes. Pharmacokinetic–pharmacodynamic studies should quantify parent compound and metabolites in plasma, kidney regions, and target cells, while formulation studies should compare micelles, lipid nanoparticles, and renal-targeted delivery systems. Finally, long-term toxicology, safety under impaired renal function, and interaction studies with cisplatin and standard renoprotective therapies are required before staged clinical evaluation.

## 10. Conclusions

Esculetin is a structurally defined natural coumarin with pharmaceutical potential as a multi-target renoprotective candidate. Current preclinical studies show model-specific improvements in cisplatin-induced AKI, diabetes complicated by ischemia–reperfusion-induced AKI, and adenine-induced chronic renal injury, together with changes in redox defense, inflammation, mitochondrial quality control, regulated cell death, and fibrotic signaling. However, these findings remain insufficient for clinical use because the evidence base is small and heterogeneous, direct targets are uncertain, oral bioavailability is low, conjugative metabolism is rapid, and long-term safety has not been established. The next phase of development should prioritize causal target validation, standardized therapeutic studies, renal exposure and metabolite analysis, formulation or targeted-delivery optimization, and regulatory-grade toxicology. Clinical trials should be considered only after these pharmacokinetic, pharmacodynamic, and safety barriers have been resolved.

## Figures and Tables

**Table 1:** Summary of experimental studies evaluating esculetin in kidney injury models.

Experimental Model	Esculetin Dose	Administration Route and Timing	Evaluated Biomarkers or Readouts	Reported Mechanisms	Main Findings and Critical Limitations
Cisplatin-induced AKI in male C57BL/6 mice	40 mg/kg/day	Oral gavage for 4 days before cisplatin challenge	SCr, BUN, histology, NGAL, KIM-1, cytokines, chemokines, adhesion molecules, MDA, 4-HNE, GSH, UPR markers, TUNEL, p53, cleaved caspase-3, PTGS2, ACSL4, TFR1, GPX4, and SLC7A11	Inflammation, oxidative stress, ER stress, apoptosis, and ferroptosis	Reduced renal dysfunction, tubular injury, oxidative stress, inflammation, ER stress, apoptosis, and ferroptosis markers. Limitations: one preventive dose, male mice, no causal rescue, and no long-term outcomes [29].
Type 1 diabetic male Wistar rats with bilateral renal ischemia–reperfusion injury	50 or 100 mg/kg/day	Oral pretreatment for 5 days before ischemia–reperfusion injury	Plasma creatinine, BUN, KIM-1, oxidative stress, mitochondrial membrane potential, PINK1, Parkin, LC3B, and p62	Mitochondrial quality control and PINK1/Parkin-associated mitophagy	Improved renal injury and mitophagy-associated readouts. Limitations: diabetic comorbidity and no causal validation of mitophagic flux [31].
Type 1 diabetic rats and high-glucose/hypoxia-reoxygenation tubular cells treated with esculetin plus phloretin	Esculetin 50 mg/kg/day with phloretin 50 mg/kg/day; 50 micromolar each in vitro	Oral pretreatment for 4 days and 1 h before surgery; combination exposure in vitro	Renal function, tubular injury, oxidative stress, inflammatory markers, mitochondrial dysfunction, and mitophagy-associated proteins	Mitophagy and inflammatory regulation	Improved AKI-diabetes comorbidity endpoints. Limitation: the effect of esculetin cannot be separated from phloretin [32].
Adenine-induced chronic renal injury and fibrotic progression in mice	30 or 60 mg/kg/day	Oral co-administration during a 4-week 0.2% adenine diet	SCr, BUN, histology, collagen deposition, inflammatory cytokines, oxidative stress markers, E-cadherin, alpha-SMA, and phosphorylation of EGFR, SRC, PI3K, AKT, and p65	EGFR/SRC/PI3K/AKT/NF-kappaB-associated inflammation and fibrosis	Attenuated renal dysfunction, inflammation, oxidative stress, and fibrosis. Limitation: the model represents crystal-associated chronic tubulointerstitial injury [30].

## Data Availability

No new data were created or analyzed in this study. Data sharing is not applicable to this article.

## Abbreviations

The following abbreviations are used in this manuscript:

AKI	Acute Kidney Injury
ARE	Antioxidant Response Element
BUN	Blood Urea Nitrogen
CKD	Chronic Kidney Disease
DN	Diabetic Nephropathy
ECM	Extracellular Matrix
EMT	Epithelial–Mesenchymal Transition
ESRD	End-Stage Renal Disease
GFR	Glomerular Filtration Rate
H2O2	Hydrogen Peroxide
HO-1	Heme Oxygenase-1
HUA	Hyperuricemia
IRI	Ischemia–Reperfusion Injury
Keap1	Kelch-Like ECH-Associated Protein 1
KIM-1	Kidney Injury Molecule-1
LN	Lupus Nephritis
MDA	Malondialdehyde
NF-κB	Nuclear Factor-κB
NLRP3	NLR Family Pyrin Domain Containing 3
Nrf2	Nuclear Factor Erythroid 2-Related Factor 2
NQO1	NAD(P)H:Quinone Oxidoreductase 1
PINK1	PTEN-Induced Putative Kinase 1
RCTs	Randomized Controlled Trials
ROS	Reactive Oxygen Species
SCr	Serum Creatinine
SOD	Superoxide Dismutase
TGF-β1	Transforming Growth Factor-β1
TNF-α	Tumor Necrosis Factor-α
UUO	Unilateral Ureteral Obstruction
